# Screening for common eye diseases in the elderly with Optos ultra-wide-field scanning laser ophthalmoscopy: a pilot study with focus on ocular toxoplasmosis

**DOI:** 10.1007/s10792-020-01683-z

**Published:** 2021-03-16

**Authors:** Pablo Eduardo Logroño Wiese, Frank Seeber, Anne-Sophie Endres, Claudia Brockmann, Uwe Pleyer

**Affiliations:** 1 Department of Ophthalmology, Charité, University Hospital Berlin, Campus Virchow, Augustenburger Platz 1, Berlin, 13353 Germany; 2 FG 16: Mycotic and Parasitic Agents and Mycobacteria, Robert Koch Institute, Seestraße 10, Berlin, 13353 Germany; 3 Evangelisches Geriatriezentrum Berlin, Reinickendorfer Str. 61, Berlin, 13347 Germany; 4 Department of Ophthalmology, Universitätsmedizin Rostock, Doberaner Str. 140, 18055 Rostock, Germany

**Keywords:** Geriatric screening, Ocular toxoplasmosis, *T. gondii*, Ultra-wide-field scanning laser ophthalmoscopy

## Abstract

**Purpose:**

Studies on the occurrence of ocular toxoplasmosis (OT) in a general population are rare. Therefore, we conducted this pilot study to assess whether a nonmydriatic ultra-wide-field (UWF) scanning laser ophthalmoscope (SLO) is suitable for a simple, rapid screening procedure.

**Methods:**

The population of this cross-sectional study was randomly recruited from a cohort of hospital-based patients in an urban geriatric hospital. Ophthalmologic evaluation was performed on 201 eyes from 101 participants through nonmydriatic UWF-SLO (Optos Daytona) and assessed for suspicious lesions and other relevant ocular findings. All images were evaluated by two independent examiners. Individuals who presented lesions with a morphological appearance suggestive of OT underwent fundoscopy and serological analysis of *Toxoplasma gondii*-specific antibodies.

**Results:**

The mean age of the study group was 76 years, and 63 (62%) were female. Despite many health restrictions, the SLO examination was carried out easily in this geriatric population. Three participants presented findings by SLO suspicious for *T. gondii*-related injury. Further clinical examination and serological investigation confirmed the diagnosis, with funduscopic evaluation and positive *T. gondii* ELISA testing. In addition, a high rate of arterial hypertension and dyslipidemias within the cohort led to a high incidence of vascular changes and age-related fundus findings.

**Conclusion:**

In our study, we confirm that UWF-SLO technology is helpful in the rapid detection of peripheral retinal injuries in elderly patients such as OT and may be used as a routine screening tool.

## Introduction

*Toxoplasma gondii* is a highly successful opportunistic parasite, which can be successfully treated upon detection, despite infecting approximately one-third of the world’s population [1]. Most primary infections in humans remain asymptomatic; however, target organs such as the eyes and brain are prone to sequelae which may affect daily function. Clinically relevant manifestations include congenital toxoplasmosis, systemic toxoplasmosis with neurological involvement (encephalitis) and ocular toxoplasmosis (OT). OT is in fact documented as the most common cause of posterior uveitis, responsible for 30% to 50% of cases in immunocompetent individuals [2].

The prevalence of *T. gondii* infections varies greatly between different countries [3–5]. For example, a low seroprevalence has been reported for North America (22.5%) [6, 7], while figures as high as 50% to 80% have been reported for South America [8]. Moreover, while the conversion rate has risen in several regions of the world, a decline in seroconversion has been observed in several nations, such as France [9], the Netherlands [10] and the USA [6, 7, 11]. Furthermore, Germany has a seroprevalence of 50%, figures that in an international context should be regarded as a relevant threat to public health [12]. Remarkably, recent data indicate not only that seroprevalence increases with age [8, 13, 14] but that gender and body mass index may correlate as potential risk factors for seropositivity [10].

The incidence of OT varies with diverse geographic seroprevalence patterns in humans. Accordingly, it is much higher in South America, where the prevalence of OT was shown to be as high as 17.7% [8], in a study from southern Brazil [14]. A retrospective study in Germany reported that 4.2% of all the uveitis cases in their referral center were attributed to infection by *T. gondii* [15]. A study in Great Britain [16] describing phenotypical differences in cases of OT reported that patients with a positive IgM test were on average much older (51.1 ± 15.3 years) than those with a negative IgM serology (34.1 ± 11.8 years).

Although OT is a major cause of posterior uveitis in many countries, reliable epidemiological data are rare. *T. gondii* can be transmitted from mother to fetus, frequently resulting in chronic recrudescent *T. gondii* retinochoroiditis. However, the vast majority of OT is considered to be acquired after birth [2, 17], mostly through the consumption of raw or undercooked meat products [18, 19] which contain *T. gondii* cysts within the tissue. Following infection, most individuals show limited symptoms without major health consequences. Nevertheless, neuronal tissue is vulnerable, and its cells are the main target of the parasite. Despite the fact that it remains unclear which route conduces this organism to the retina, ocular infection is one of the major manifestations in humans.

Necrotizing retinochoroiditis is considered the hallmark presentation of OT. It is so characteristic that in the clinical routine no further laboratory workup is required since the diagnosis is made on a clinical basis [20]. Interestingly, more than 70% of OT patients visiting an ophthalmic center in the Netherlands presented with a combination of an active lesion and a healed retinal scar [21]. This observation may imply that previously occurring retinitis remained unnoticed. Nonetheless, it remains speculative whether first manifestations follow a less severe course than recurrent episodes of illness. Whereas all of these data are derived from ophthalmic centers, information on real-world data is scarce. Altogether, it is not yet clear how many individuals are eventually affected by previous OT, since visual disturbances may be limited and therefore underreported due to the relatively silent course of the disease. In addition, retinal lesions may even be overlooked on routine ocular exams because of their peripheral localization on the retinal margins. The ultra-wide-field (UWF) scanning laser ophthalmoscopy (SLO) has been introduced as a fundus imaging technology, which allows nonmydriatic fundus photograph while also capturing a 180° to 200° degree perspective of the retinal surface with vivid resolution [22]. This technology enables an accurate characterization of ocular lesions at almost every point on the retina. This can be achieved with a single high-resolution image (Optomap) showing detailed features across the posterior pole and peripheral retina.

The large burden on health care systems for OT is illustrated by an estimated 250,000 patient visits to ophthalmologists each year in the USA alone [23]. Recent data estimate the occurrence of ocular lesions due to toxoplasmosis in 21,505 people per year in the USA, where between 2,150 and 7,527 individuals per year may develop symptomatic cases of OT [24]. Thus, the present investigation was initiated as a pilot project for the future assessment of the occurrence of OT.

## Material and methods

### Sample characteristics

This investigation was designed as a cross-sectional, single-site, clinic-based study to evaluate the presence of OT lesions. One hundred and one consecutive individuals were recruited for examination from the “Evangelisches Geriatriezentrum Berlin” between August and October 2016. The vast majority of participants examined were over the age of 70, most were women (63) (Table 1). The sample was not designed, but rather chosen randomly in an open recruitment process. Screening subjects were informed about the aims and methods of performing the screening examinations and written consent was obtained. There were no exclusion criteria to this investigation, as it entails a noninvasive, nonmydriatic UWF-SLO examination. A total of 201 eyes were scanned (one participant had an eye prosthesis).

**Table 1:** Sample demographics: age and gender distribution (*n* = 101)

Age group:	Participants (101)	Male (38)	Female (63)
< 50	2 (1.98%)	2 (1.98%)	0 (0.00%)
50–59	6 (5.94%)	2 (1.98%)	4 (3.96%)
60–69	8 (7.92%)	2 (1.98%)	6 (5.94%)
70–79	43 (42.57%)	21 (20.79%)	22 (21.78%)
80–89	38 (37.62%)	9 (8.91%)	29 (28.71%)
> 90	4 (3.96%)	2 (1.98%)	2 (1.98%)

### Ultra-wide-field imaging and data analysis

A Daytona ultra-wide-field retinal camera (Optos PLC, Dunfermline, UK) was utilized, which is based on UWF-SLO technology and captures fundoscopic images within less than 0.4 s. Retinal scanning was performed by one of the authors (PELW). Image data were stored for further descriptive analysis of each fundus image. Fundus documentation was carried out by capturing several images of each eye until the most ample diagnostic field was achieved in both color and autofluorescence mode for both eyes. Total examination time varied with patient positioning, but despite seldom impediments to optimal image acquisition, examination time remained under 7 min per person. After an eye-oriented anamnesis, slit lamp examination was used to detect media opacity. Fundus scanning was performed at least twice per eye in both color and autofluorescence mode. In cases of severe ptosis, an ophthalmic cotton swab was used to aid the eyelid in order to avoid suboptimal diagnostic images. After systematic interpretation of the resulting images a report was noted for every eye, each classified per anatomic region and presumptive diagnosis. Based upon this data collection, a descriptive exploratory analysis was carried out by calculating the simple frequency of repeating conditions. Having entered the demographic data of the total population into the sample spreadsheet, a segmentation was made by clinical pathological findings, later regrouped by a presumptive diagnosis and classified according to age group, gender, eye and anatomic location within the eye. All images were evaluated by two independent examiners.

### Serological testing

Participants who presented a fundus lesion suspicious for a *T. gondii*-related activity were made aware of this finding and a blood sample was collected for further serological analysis. Serum samples were tested for the presence of anti-*T. gondii* IgG antibodies using a commercial ELISA (EUROIMMUN AG, Lübeck, Germany) according to the manufacturer’s protocol.

## Results

### Ocular toxoplasmosis detection through UWF-SLO

**Fig. 1:**
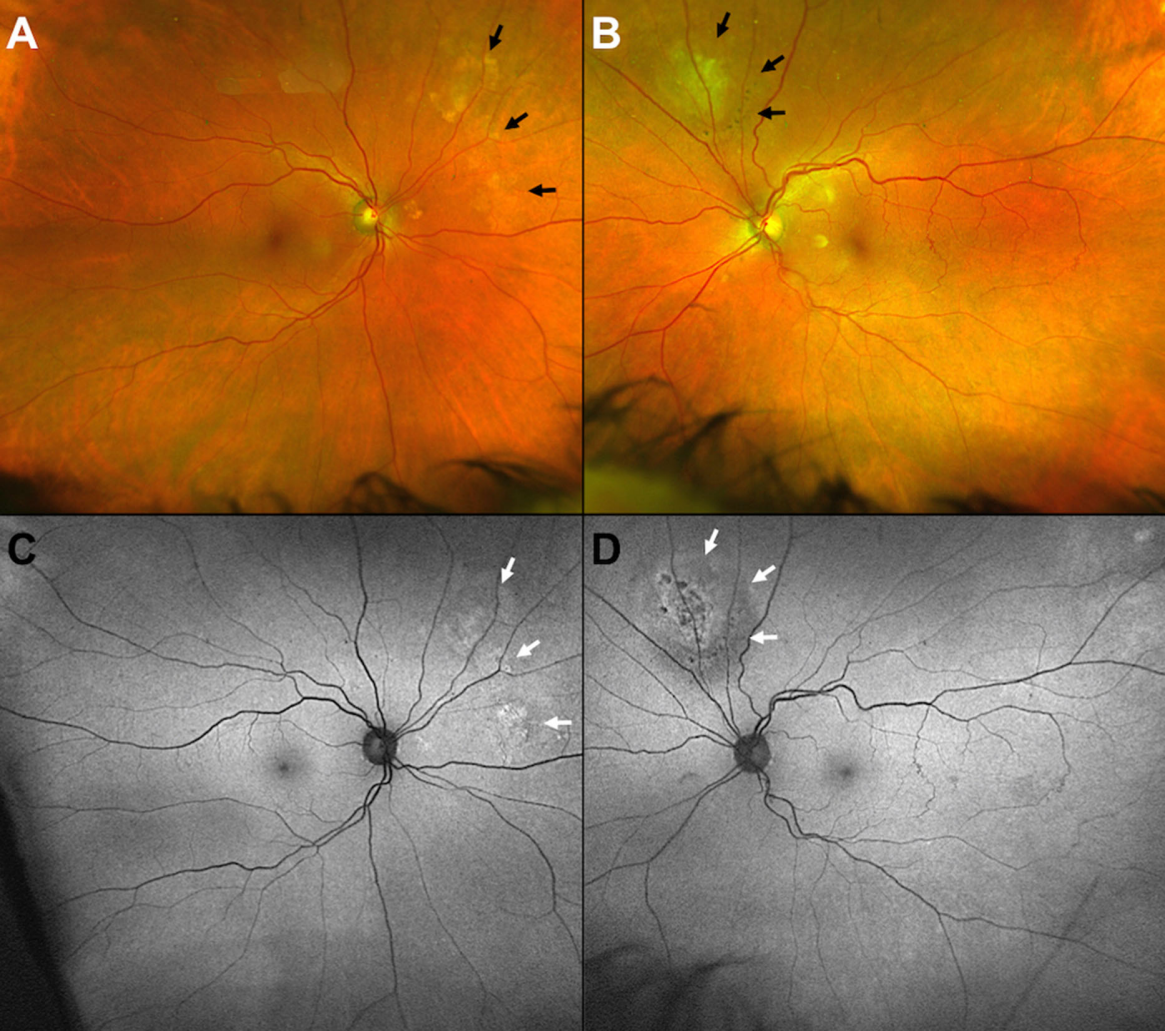
Clinical appearance of toxoplasmic retinochoroiditis. **a** Superonasal perivascular lesions in proximity to the optic disk, **b** superopapillary chorioretinal lesion with pigmentary changes and signs of cicatrization; **c** and **d** fundus autofluorescence of the corresponding lesions revealing the true extent of the underlying chorioatrophic injury (arrows)

A total of 201 eyes were examined in this study. The convenience of rapid, high-quality image capture enabled the examination of all participants, despite limitations due to obesity, motion limitations, spinal column disorders or chronic oxygen requirement. In order to find a case of OT in asymptomatic participants, certain key characteristics of the typical OT presentation were taken into consideration, to avoid overlooking a positive case. Typically, OT lesions present as a whitening or yellowing patch of retinochoroiditis (Fig. 1), usually perivascular, and often associated with pigment changes in the retina (Fig. 1d) [2]. In order to minimize false negatives, the best possible diagnostic field was obtained since lesions may occur anteriorly, even close to the ora serrata and remain unnoticed at first inspection [2]. Therefore, several scans were performed in some individuals to achieve optimal diagnostic images.

Three out of 101 individuals presented lesions attributable to the *T. gondii* parasite. One participant showed asymmetric bilateral lesions, while the other two presented with unilateral manifestations. A stark contrast was found when comparing the apparent extension of OT lesions on a color fundus against the autofluorescence image of the same eye (Fig. 1). The rapid on-site assessment of each Optomap image for suspicious OT lesions was key in the detection. These three individual were subsequently revisited by a senior ophthalmologist (UP) using indirect ophthalmoscopy for reevaluation, and OT lesions were confirmed in all cases. In addition, all suspects for OT were tested by Toxo-IgG ELISA and were highly positive (> 200 IU) at a 1:100 serum dilution. It remains unclear whether or not these cases were a recurrence or a more recent infection. However, none of the participants showed other clinical findings such as blurred edges of the lesions or vitreous haze typical for OT activity. In addition, none referred any current or past symptoms. Given that there were no signs of acute infection at the time of examination, further investigations such as an Ig-M test or an aqueous humor tap were not indicated.

### Other findings

As can be expected from an older cohort of subjects, a number of other, primarily age-related changes were observed. Systemic arterial hypertension affected at least 68 (67%) of the study participants (Table 2). 45.3% of the eyes examined showed vascular characteristics indicative of hypertensive changes. Forty participants suffered from dyslipidemia, mostly hypercholesterolemia. Thirty-two study participants were subject to chronic kidney insufficiency. Type 2 diabetes mellitus affected 26 (26%) individuals within our cohort, and 5 (5%) of the eyes examined showed signs of diabetic alterations. Eleven participants were clinically obese, many of which suffered from hepatic steatosis. Table 2 illustrates various systemic diseases affecting some of the participants, including neurologic, endocrine, cardiopulmonary, autoimmune and oncologic conditions. A detailed listing of these changes is not the focus of this study; therefore, only few examples were selected in the context of this screening approach.

**Table 2:** Identified comorbidities based on clinical history: systemic risk factors (*n* = 101)

Comorbidities: (DSM-Code)	Participants (101)	Male (38)	Female (63)
Cardiovascular			
(I10) Arterial hypertension	68 (67.33%)	23 (22.77%)	45 (44.55%)
(I25) Ischemic heart disease	25 (24.75%)	15 (14.85%)	10 (9.90%)
(I34-37) Valvular heart disease	28 (27.72%)	10 (9.90%)	18 (17.82%)
(I44-45) Atrioventricular block	17 (16.83%)	9 (8.91%)	8 (7.92%)
(I48) Atrial fibrillation	26 (25.74%)	15 (14.85%)	11 (10.89%)
(I50) Heart failure	24 (23.76%)	13 (12.87%)	11 (10.89%)
(I73) Peripheral arterial occlusive disease	6 (5.94%)	2 (1.98%)	4 (3.96%)
(F17) Nicotine dependence	12 (11.88%)	6 (5.94%)	6 (5.94%)
(E11) Diabetes mellitus	26 (25.74%)	12 (11.88%)	14 (13.86%)
(E66) Obesity	11 (10.89%)	5 (4.95%)	6 (5.94%)
(E78) Dyslipidemia	40 (39.60%)	15 (14.85%)	25 (24.75%)
Renal			
(N18) Chronic kidney disease	32 (31.68%)	15 (14.85%)	16 (15.84%)
Inflammatory			
(L40) Psoriasis	1.98% (2)	1 (0.99%)	1 (0.99%)
(M06) Rheumatoid arthritis	6 (5.94%)	1 (0.99%)	5 (4.95%)
Neurologic			
(G12) Amyotrophic lateral sclerosis	1 (0.99%)	0 (0.00%)	1 (0.99%)
(G20) Parkinson’s disease	3 (2.97%)	1 (0.99%)	2 (1.98%)
(G70) Myasthenia gravis	1 (0.99%)	1 (0.99%)	0 (0.00%)
(G81) Hemiparesis	11 (10.89%)	4 (3.96%)	7 (6.93%)
(I63) Cerebral infarction	28 (27.72%)	13 (12.87%)	15 (14.85%)
(I65) Carotid artery stenosis	5 (4.95%)	1 (0.99%)	4 (3.96%)
Oncologic			
(N42) Prostatic carcinoma	1 (0.99%)	1 (0.99%)	0 (0.00%)
(C50) Mammary carcinoma	2 (1.98%)	0 (0.00%)	2 (1.98%)
(C34) Lung carcinoma	2 (1.98%)	0 (0.00%)	2 (1.98%)
(Z85) Colon carcinoma	3 (2.97%)	1 (0.99%)	2 (1.98%)
(C43) Skin melanoma	2 (1.98%)	1 (0.99%)	1 (0.99%)
(C44) Squamous cell carcinoma	3 (2.97%)	1 (0.99%)	2 (1.98%)
(C83) B cell lymphoma	1 (0.99%)	0 (0.00%)	1 (0.99%)

#### Vascular anomalies

**Fig. 2:**
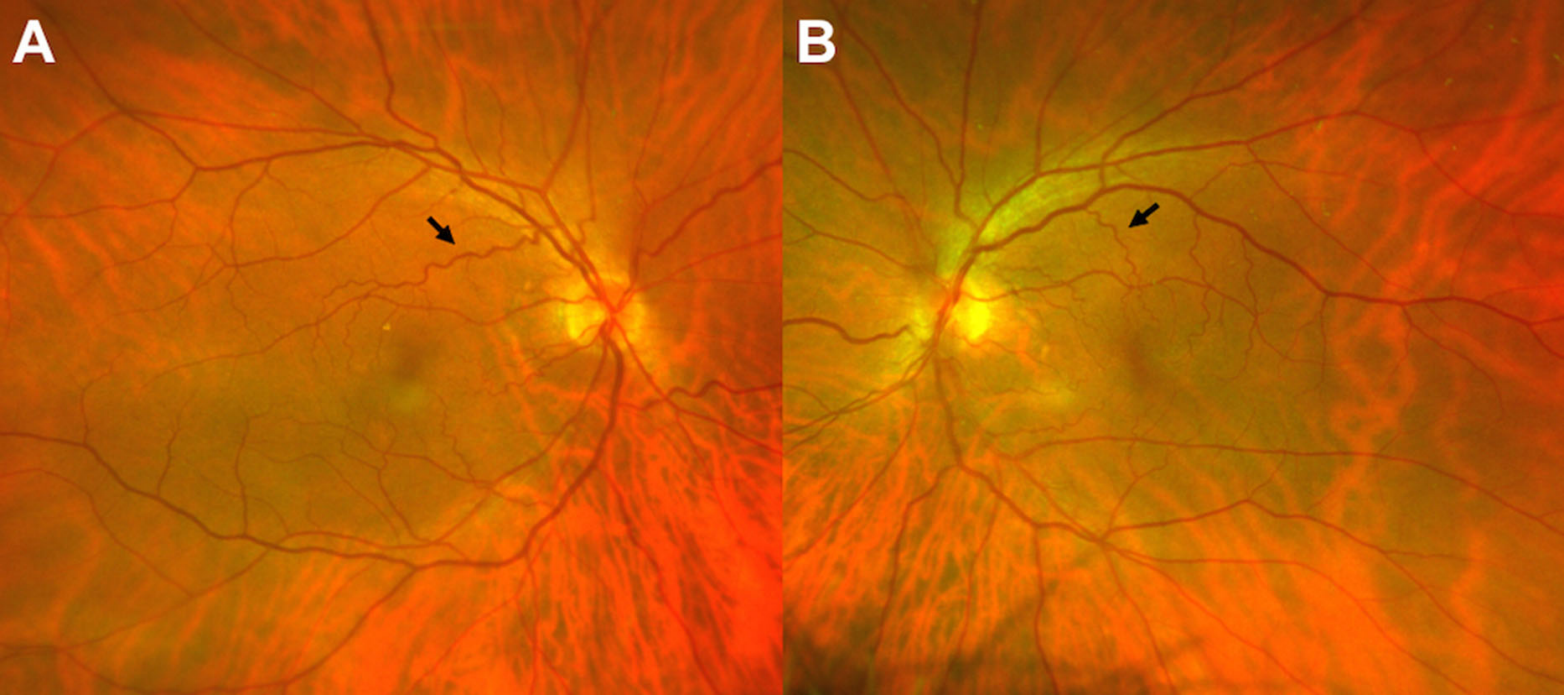
Wide-field fundus photographs (amplified) of a participant with severe undiagnosed bilateral carotid atheromatosis, showing marked venous tortuosity (arrows), venous beading and cotton wool spots

A 63-year-old male participant presented with suspicious vascular tortuosity, venous beading and cotton wool spots (Fig. 2). Since he admitted to being a regular smoker and had not been medically checked for many years, he was referred for further cardiovascular control. A Doppler ultrasound examination revealed severe bilateral carotid sinus occlusion, condition for which he was subsequently managed through endarterectomy.

**Fig. 3:**
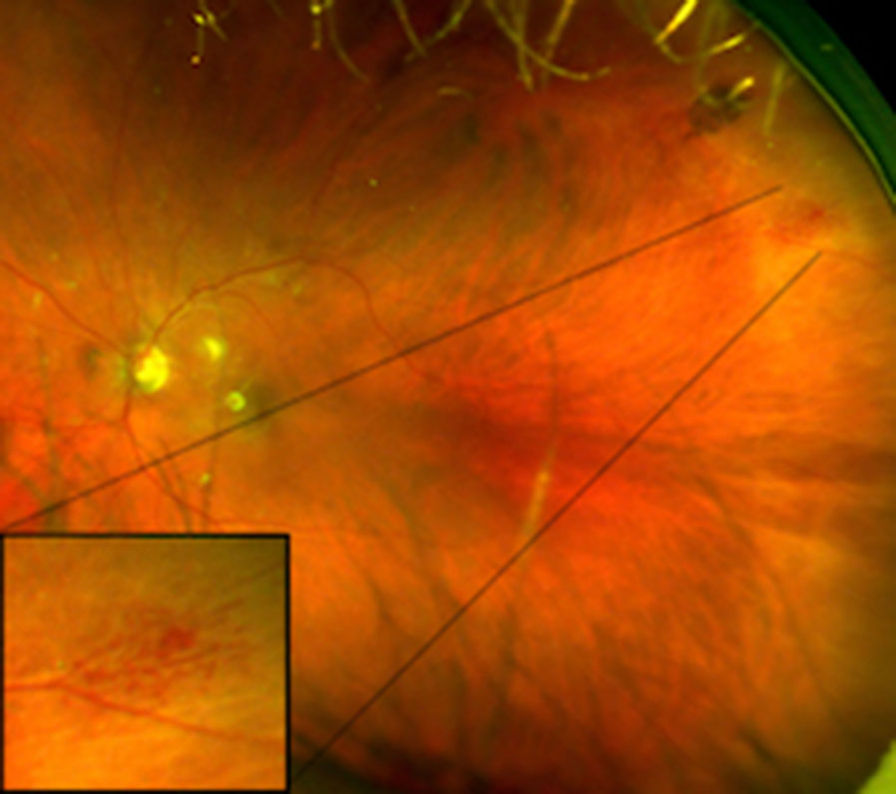
Wide-field fundus photograph showing peripheral neovascularization (amplification) in a diabetic participant

A 75-year-old diabetic participant presented with an unidentified seafan-shaped vasoproliferative lesion (Fig. 3), calling for focal laser photocoagulation, on the superotemporal periphery of his left eye. Overall, various hemorrhages were identified in 29 (14.4%) of the eyes examined (Table 3), while branch retinal vein occlusion (BRVO) was recognized in 3 (1.5%) of the eyes examined.

**Table 3:** Clinical findings in the posterior segment based on anatomic location. *n* = 201

	Participants (101)	Male (38)		Female (63)	
Fundoscopic findings:	Eyes (201)	OD (38)	OS (37)	OD (63)	OS (63)
*Vitreous*					
Asteroid hyalosis	3 (1.49%)	1 (0.50%)	0 (0.00%)	2 (1.00%)	0 (0.00%)
Floaters	68 (33.83%)	12 (5.97%)	10 (4.98%)	21 (10.45%)	25 (12.44%)
*Optic nerve head*					
Bergmeister’s papilla	1 (0.50%)	0 (0.00%)	0 (0.00%)	1 (0.50%)	0 (0.00%)
Myelinated fibers	1 (0.50%)	0 (0.00%)	0 (0.00%)	1 (0.50%)	0 (0.00%)
Hemorrhage	3 (1.49%)	0 (0.00%)	0 (0.00%)	1 (0.50%)	2 (1.00%)
Tilted disk	8 (3.98%)	1 (0.50%)	2 (1.00%)	1 (0.50%)	4 (1.99%)
Peripapillary atrophy	54 (26.87%)	7 (3.48%)	8 (3.98%)	20 (9.95%)	19 (9.45%)
Elevated cup-to-disk ratio	86 (42.79%)	20 (9.95%)	19 (9.45%)	24 (11.94%)	23 (11.44%)
Glaucoma suspect	56 (27.86%)	13 (6.47%)	12 (5.97%)	15 (7.46%)	16 (7.96%)
*Macula*					
Posterior staphyloma	4 (1.99%)	0 (0.00%)	0 (0.00%)	2 (1.00%)	2 (1.00%)
Epiretinal membrane	8 (3.98%)	3 (1.49%)	3 (1.49%)	1 (0.50%)	1 (0.50%)
Macular hole	1 (0.50%)	0 (0.00%)	0 (0.00%)	0 (0.00%)	1 (0.50%)
Hard exudates	1 (0.50%)	0 (0.00%)	0 (0.00%)	1 (0.50%)	0 (0.00%)
Cotton wool spots	1 (0.50%)	0 (0.00%)	0 (0.00%)	0 (0.00%)	1 (0.50%)
Hemorrhage	4 (1.99%)	0 (0.00%)	1 (0.50%)	0 (0.00%)	3 (1.49%)
Macular edema	6 (2.99%)	1 (0.50%)	1 (0.50%)	1 (0.50%)	3 (1.49%)
Pigment displacement	44 (21.89%)	4 (1.99%)	3 (1.49%)	18 (8.96%)	19 (9.45%)
Drusen	39 (19.40%)	6 (2.99%)	5 (2.49%)	13 (6.47%)	15 (7.46%)
Macular degeneration	18 (8.96%)	2 (1.00%)	3 (1.49%)	6 (2.99%)	7 (3.48%)
Geographic atrophy	9 (4.48%)	2 (1.00%)	4 (1.99%)	2 (1.00%)	1 (0.50%)
*Blood vessels*					
Branch retinal vein occlusion	3 (1.49%)	2 (1.00%)	0 (0.00%)	0 (0.00%)	1 (0.50%)
Hypertensive retinopathy	91 (45.27%)	17 (8.46%)	17 (8.46%)	28 (13.93%)	29 (14.43%)
Vascular tortuosity	43 (21.93%)	9 (4.48%)	12 (5.97%)	11 (5.47%)	11 (5.47%)
Arteriolar narrowing	72 (35.82%)	13 (6.47%)	17 (8.46%)	22 (10.95%)	21 (10.45%)
Arteriovenous nicking	26 (12.94%)	4 (1.99%)	5 (2.49%)	8 (3.98%)	9 (4.48%)
Copper wire arterioles	4 (1.99%)	1 (0.50%)	0 (0.00%)	2 (1.00%)	1 (0.50%)
Silver wire arterioles	12 (5.97%)	2 (1.00%)	3 (1.49%)	1 (0.50%)	6 (2.99%)
Venous beading	6 (2.99%)	1 (0.50%)	1 (0.50%)	2 (1.00%)	2 (1.00%)
Diabetic retinopathy	5 (2.49%)	2 (1.00%)	2 (1.00%)	0 (0.00%)	1 (0.50%)
Neoproliferative seafan	1 (0.50%)	0 (0.00%)	1 (0.50%)	0 (0.00%)	0 (0.00%)
*Peripheral retina*					
Toxoplasmosis lesion	4 (1.99%)	0 (0.00%)	0 (0.00%)	3 (1.49%)	1 (0.50%)
Photocoagulation scar	6 (2.99%)	1 (0.50%)	1 (0.50%)	0 (0.00%)	4 (1.99%)
Chorioretinal atrophy	15 (7.46%)	3 (1.49%)	4 (1.99%)	2 (1.00%)	6 (2.99%)
Chorioretinal nevus	13 (6.47%)	4 (1.99%)	3 (1.49%)	4 (1.99%)	2 (1.00%)
Drusen	40 (19.90%)	5 (2.49%)	5 (2.49%)	13 (6.47%)	17 (8.46%)
Hemorrhage	22 (10.95%)	3 (1.49%)	10 (4.98%)	5 (2.49%)	4 (1.99%)
Cobblestone degeneration	9 (4.48%)	1 (0.50%)	1 (0.50%)	3 (1.49%)	4 (1.99%)

#### Vitreoretinal interface anomalies

Epiretinal membranes (ERM) may develop in cases of intense vitritis [2]. ERM were found in 8 (3.9%) of the eyes examined using UWF-SLO. A full thickness macular hole was observed in one of the study participants. Asteroid hyalosis was found in 3 (1.5%) eyes, looking remarkable through UWF-SLO. Sixty-eight (33.8%) eyes (Table 3) had floaters.

#### Optic nerve anomalies

All Optomaps were evaluated for optic nerve color, border definition, cup-to-disk ratio and aspect of the vasculature. Eighty-six (42.8%) of the eyes showed an elevated cup-to-disk ratio, leading to loss of the neuroretinal rim area and nasal rejection of the retinal vessels. Signs suspicious for glaucomatous change were found in 56 (27.9%) of the eyes. Peripapillary atrophy was identified in 54 (26.9%) eyes. In one participant with a prior history of poliomyelitis, an extensive peripapillary and parafoveolar retinochoroidal atrophy was shown.

#### Macular degeneration

Given the average age of the study group and considering that most of the participants were light-skinned Europeans [25], signs of age-related macular degeneration (AMD) were repeatedly identified among the participants. Manifest AMD was identified in 18 (9%) eyes, and 9 eyes (4.5%) presented with geographic atrophy. Early signs of AMD, such as macular drusen, were identified in at least 39 (19.4%) of the eyes examined.

#### Peripheral retina anomalies

Some retinal lesions were readily detectable, even with just a color image. Drusen [26] were easily identified scattered peripherally across all quadrants in 40 (19.9%) of the eyes examined (Table 3). Cobblestone degeneration (4.5%), chorioretinal atrophy (7.5%) and nevi (6.5%) were easily identified in several eyes. Six (3%) eyes had been previously treated with laser photocoagulation.

## Discussion

Very few studies have reported on the occurrence and frequency of retinal lesions produced by OT in the general population, and even rarer are reports in an older population. To our knowledge, this is the first publication specifically focusing on the detection of OT using UWF-SLO. It is important to emphasize that OT remains essentially a clinical diagnosis. This is due to the fact that ocular lesions remain often asymptomatic, which relates to the location at the retinal periphery and therefore might be easily overlooked. Indeed, all three of our positive findings were detected on the mid-peripheral retina. One participant had bilateral involvement, which corresponds to findings in previous German studies, where 61% of patients had bilateral ocular lesions [27]. In general, the UWF-SLO system made a decisive contribution to our screening procedure. Of all the systems available so far, its 200º degree view allows the most extensive assessment of the peripheral retina to date. It has also proved very practical with our geriatric cohort. Without any further preparation, all of our test subjects could be examined quickly and easily without exception despite a wide range of age-related ailments. Since the Optomaps were immediately available, a quick follow-up search for suspicious lesions could be carried out in individual cases. The autofluorescence mode was particularly useful in detecting the real extension of certain retinal lesions, which sometimes seemed less extensive or even inexistent in the classic color fundus image (Fig. 1). The fact that subjacent choroidal injury can appear subclinical on color fundus photograph and typical fundus ophthalmoscopy (Fig. 1a) may result in cases where early retinochoroidal injury remains undetected, especially when located further from the posterior pole. The light scattering effect of melanin in the RPE reduces the blue light reflected, as it predominantly absorbs the lower-wavelength colors of the spectrum, giving a more intense yellow and red shift to a lesion appearance [26], blending it with the overlying tissue. Another advantage, particularly in our elderly individuals, was that this mode of image capture proved useful even in the presence of media opacity of the lens [28] or the vitreous. Several participants presented with cataracts, which sometimes constituted an obstacle to achieving optimal diagnostic images. Using the Daytona’s eye steering option was definitely helpful in identifying peripheral lesions and sometimes key in achieving acceptable resolution images in the presence of lens opacification. In order to further secure our UWF-SLO findings, both a detailed fundoscopy and a serological confirmation of the affected persons were subsequently carried out.

It is also important to emphasize that OT rates of seropositivity do not mirror rates of the clinical disease. Based on our assessment of clinically silent ocular lesions, our data correspond to previous calculations of OT prevalence. Before starting this research, a conservative prediction based on RKI data estimated the prevalence of OT in Germany between 2 and 4%, which most accurately corresponds with our actual findings of 3%, for 3 confirmed cases. It must be emphasized that we only started this study as a pilot project, in order to gain experience on the practicability and clinical value of the UWF-SLO system, but not as an epidemiological study, since the cohort of our sample is too small for this. Very recently, another much larger (“Gutenberg”) study has reported a 0.2% prevalence of OT within its German cohort [29]. In this cross-sectional analysis of more than 15,000 subjects (aged 35–74 years) also a nonmydriatic fundus camera was used but acquiring only 30° fundus images. Therefore, more peripheral lesions, characteristic for OT, may have been easily missed. In addition, age and gender distribution differ and may further explain the lower rate of OT suspected retina lesions.

It is widely documented that the prevalence of *T. gondii* infection varies greatly across the globe, depending on the geographic region and the population studied. Most certainly, environmental factors play a role in the disease transmission. Indeed, a recent screening approach indicated a persistently high (1.1%) yearly seroconversion rate in Germany, where as many as 77% of individuals between the ages of 70 and 79 tested positive for *T. gondii* IgG antibodies [10]. Although several serological studies based on a large population have been presented, the occurrence and consequences of clinical disease have not been well documented. The rate of ocular infection in seropositive individuals may vary greatly. Based on data from Smith and Ganley [30] and Holland [31], an estimated rate of 2% results in 21,505 persons with retinal lesions due to OT per year in the USA [24]. Even higher numbers have been reported in Brazil [3], which may be related to more virulent strains of *T. gondii* in South America [8, 32, 33]. Interestingly, in previous studies the prevalence of inactive ocular lesions varies not only worldwide, but also within the same country, e.g., in Brazil, where a prevalence as high as 17.7% has been observed [8].

It must be emphasized that individuals affected from OT can eventually suffer significant vision loss and that treatment still remains limited. In addition, an association between *T. gondii* infection and psychiatric disorders has been hypothesized [34]. Since most of these data are derived from animal studies [35] and observations which are incompatible with the human situation, we are careful in overestimating these finding. None of our three OT individuals was diagnosed of any psychiatric disorder. However, in congenital T. gondii infection severe neurological manifestations may occur and have been estimated to be responsible for a cumulative 620–1200 disability-adjusted life years (DALYs) in the Netherlands [36]. Therefore, information about the burden of the disease is useful for establishing public health measures to take prevention into effect.

**Fig. 4:**
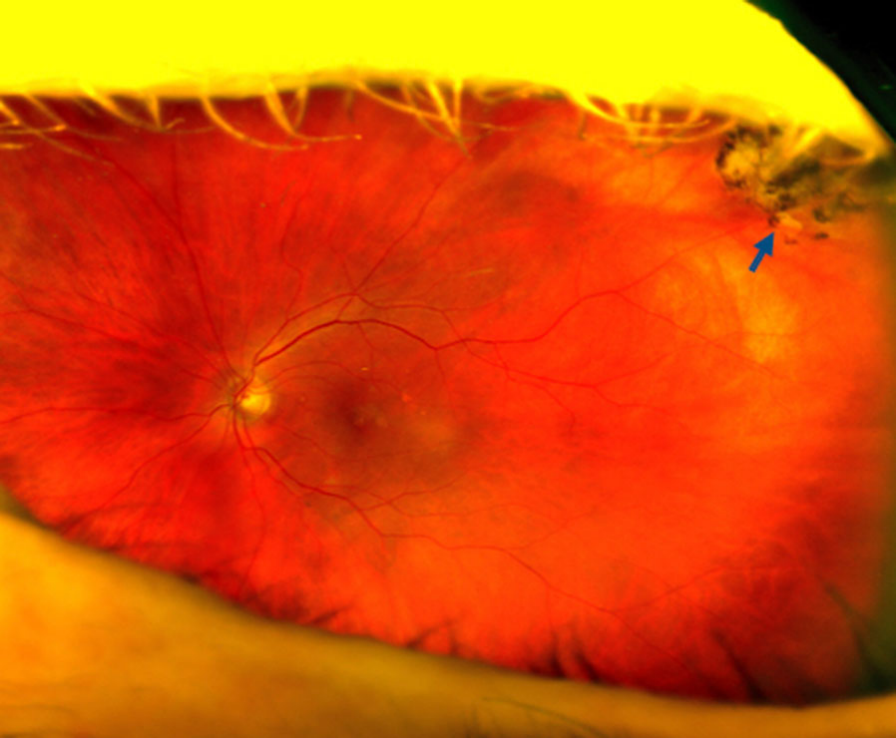
Wide-field color fundus photograph of a left eye showing a peripheral retinochoroidal scar due to focal photocoagulation (arrow)

**Fig. 5:**
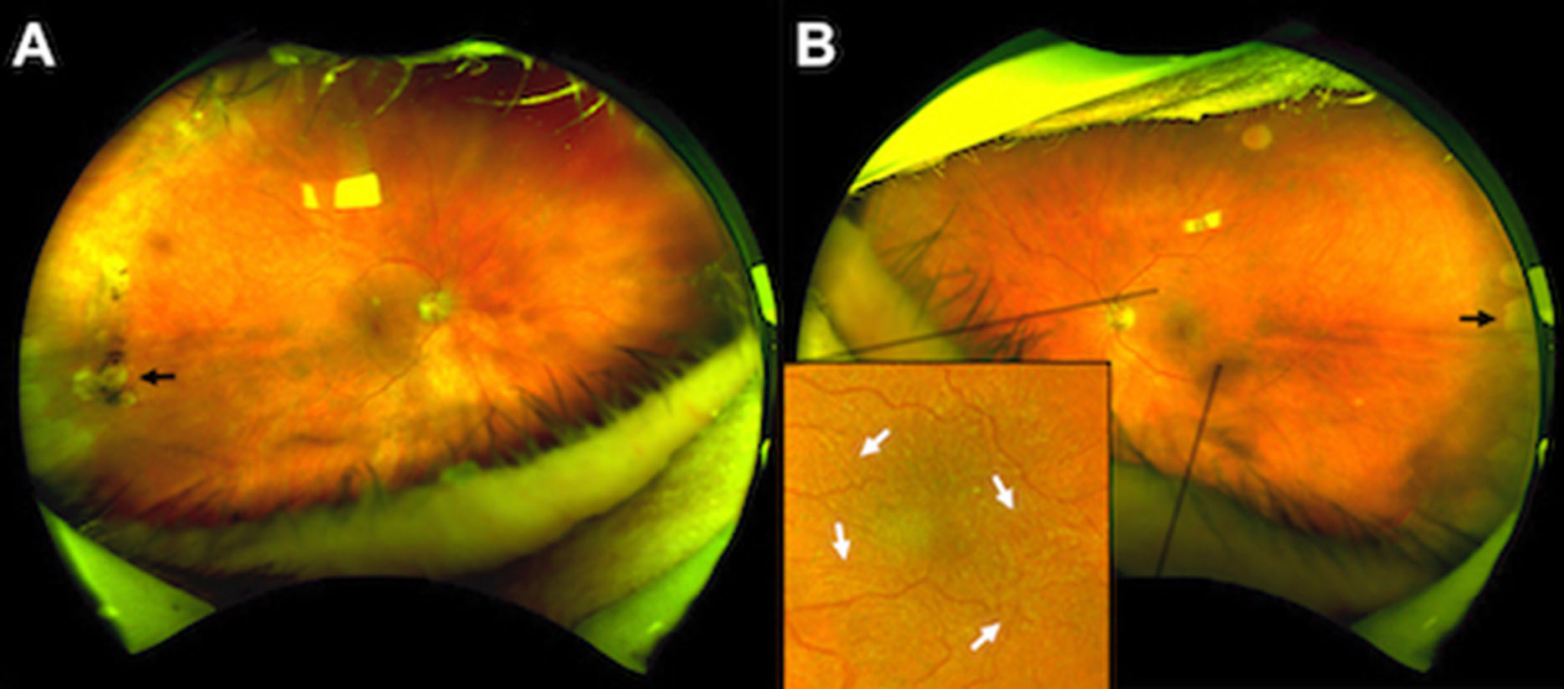
Wide-field fundus photographs of a participant with Cobblestone degeneration on both eyes (black arrows), also showing an epiretinal membrane (white arrows) in the left eye

Our investigation has several limitations. First, it was performed at a single geriatric center. Therefore, a referral bias and preselection might have influenced our data. Second, due to the pilot character of the study, the sample size of the cohort was small. Another point to consider is that we have also carried out a fundoscopy and serological examination to secure the diagnosis and used the SLO technique as a screening. Although reliable information was obtained through UWF-SLO, there are differential diagnosis which may cause difficulties in individual cases. Even though OT typically presents as an isolated focus of retinochoroiditis [3], atypical presentations may occur [37]. Several clinical features may resemble OT, and these may include retinochoroidal scars due to focal laser photocoagulation treatment (Fig. 4), cobblestone degeneration (Fig. 5) and other atrophic and pigmentary changes of the retina. To discard various infectious etiologies, other serological testing for cases of posterior uveitis include: Quantiferon-TB, plus antibodies for Borrelia burgdorferi, Treponema pallidum, varicella-zoster and Herpes Simplex Virus 1 and 2.

Despite these limitations, this is the first report describing an ocular screening for OT with UWF-SLO. We believe that our study provides not only useful data on OT in a geriatric population, but also hints for the use of UWF-SLO in many other ocular disorders. The cost of this instrumentation may currently be prohibitive for routine use. However, the fact that image acquisition is extremely simple (even in children and the elderly) and that it does not require mydriatic drops for a wide-field fundus photograph makes the UWF-SLO a practical application for screenings, not just for ocular toxoplasmosis, but for many other eye diseases.
